# Designing Age-Friendly Communities: Exploring Qualitative Perspectives on Urban Green Spaces and Ageing in Two Indian Megacities

**DOI:** 10.3390/ijerph18041491

**Published:** 2021-02-04

**Authors:** Deepti Adlakha, Mina Chandra, Murali Krishna, Lee Smith, Mark A. Tully

**Affiliations:** 1 School of Natural and Built Environment, Queen’s University Belfast, Belfast BT9 5AG, UK; 2 Atal Bihari Vajpayee Institute of Medical Sciences and Dr. Ram Manohar Lohia Hospital, New Delhi 110001, India; minasaxena@gmail.com; 3 Foundation for Research and Advocacy in Mental Health, Mysore, Karnataka 570028, India; muralidoc@gmail.com; 4 Viveka Foundation, Vajamangala, Mysore, Karnataka 570028, India; 5 The Cambridge Centre for Sport and Exercise Sciences, Anglia Ruskin University, Cambridge CB1 1PT, UK; Lee.Smith@anglia.ac.uk; 6 Institute of Mental Health Sciences, School of Health Sciences, Ulster University, Newtownabbey BT37 0QB, UK; m.tully@ulster.ac.uk

**Keywords:** urban green spaces, built environment, healthy ageing, older adults, India

## Abstract

The World Health Organization and the United Nations have increasingly acknowledged the importance of urban green space (UGS) for healthy ageing. However, low- and middle-income countries (LMICs) like India with exponential ageing populations have inadequate UGS. This qualitative study examined the relationships between UGS and healthy ageing in two megacities in India. Participants were recruited using snowball sampling in New Delhi and Chennai and semi-structured interviews were conducted with consenting participants (N = 60, female = 51%; age > 60 years; fluent in English, Hindi, or Tamil). Interviews were recorded, transcribed, translated, and analysed using inductive and thematic analysis. Benefits of UGS included community building and social capital, improved health and social resilience, physical activity promotion, reduced exposure to noise, air pollution, and heat. Poorly maintained UGS and lack of safe, age-friendly pedestrian infrastructure were identified as barriers to health promotion in later life. Neighbourhood disorder and crime constrained older adults’ use of UGS in low-income neighbourhoods. This study underscores the role of UGS in the design of age-friendly communities in India. The findings highlight the benefits of UGS for older adults, particularly those living in socially disadvantaged or underserved communities, which often have least access to high-quality parks and green areas.

## 1. Introduction

Population ageing is a dominant global demographic phenomenon of the 21st century and accelerating at a higher rate than in the past. Worldwide, the proportion of older adults (60 years or older) is expected to double by 2050 and more than triple by 2100, rising from 962 million globally in 2017 to 2.1 billion in 2050 and 3.1 billion in 2100 [1]. Low- and middle-income countries (LMICs) are experiencing exponential growth in ageing populations. In 2009, two-thirds of the world’s older persons lived in LMICs, regions that are much less prepared to deal with this shift in population dynamics compared to high-income countries (HICs) [2]. In particular, Southeast Asian countries are currently experiencing an ageing trend that is unprecedented in history. A report by the World Health Organization (WHO) Southeast Asia Regional Framework on Healthy Ageing estimated that 289 million people in the region will be aged 60 years or over by 2030 [3]. At present, India, has the largest population of older adults at 125 million, followed by Indonesia (22 million), Bangladesh (12 million) and Thailand (11 million) [1]. The proportion of older adults in India is expected to increase to 20% (315 million) by the year 2050, which would be more than the total population of the United Status [1,3]. The rising costs of health and social care associated with age-related disease and disability makes ageing research a public health priority.

An ageing population requires additional measures to prevent or reduce the burden of disease and disability, and maintain the functional ability that enables health and wellbeing among older adults, a concept described as ‘healthy ageing’ by the WHO [3,4]. Health and wellbeing of older adults is strongly linked to their home neighbourhood, where they are likely to spend more time compared to younger populations due to a range of factors including retirement or limited mobility. Urban areas, in particular, have large numbers of older inhabitants [5,6]. In India, it is expected that around 42% of the population will live in urban areas by 2030 [7]. Across LMICs, an estimated 25% of older adults are expected to be living in cities by 2050, making urban environments important determinants for healthy ageing [8,9].

The built environment exerts an important impact on health, which can either facilitate or hinder participation in activities of daily living and healthy behaviour [10,11]. Activity-friendly neighbourhoods, with easy access to destinations and services, are known to be positively associated with higher levels of physical activity and walking among older adults [12]. A feature of the built environment that has received increasing attention over the last years is the provision of urban green space (UGS). UGS can come in many forms, including city parks, gardens, playgrounds, pocket parks, large forests, residential greenery, and areas partially or fully covered by vegetation (e.g., street trees, grass, bushes) in the neighbourhood [13]. Research from HICs has demonstrated that living in neighbourhoods with more UGS is associated with higher levels of physical activity, better mental health, lower levels of stress, and reduced risk of disease [14,15].

International agencies have identified UGS interventions as a promising strategy for healthy ageing. The United Nations Sustainable Development Goal 11.7 and the WHO recommend that UGSs are key to improving the age-friendliness of neighbourhoods [16]. As older adults are more bound to their home neighbourhood, they may especially benefit from the provision of UGSs in their immediate surroundings. Living in proximity to UGS is linked to more frequent park visitation and better perceived health among older adults [6]. However, urban living often limits access to nature and can increase exposure to environmental hazards such as air, water, and noise pollution [17].

India has been experiencing rapid urbanisation since 1970, with its urban population rising from 109 million in 1971 to 377 million in 2011, a percentage increase from 19.9 to 31.6 in four decades [18]. Currently, there are 46 cities in India with populations of 1 million and above (million-plus cities) including three cities with populations of 10 million and above (megacities) [19]. By 2030, India is projected to have six cities with a population of over 10 million, and more than 100 million-plus cities [19]. The availability of UGS is decreasing in most of these cities, with many falling short of the WHO recommended norms of 9 sq. m. UGS per capita. The benefits of UGS have been widely documented, however, a majority of the findings are from HICs [6,20,21]. This limits the generalizability of the evidence to LMICs and there is minimal research exploring the influence of UGS on ageing in India. To the best of our knowledge, this is the first study to fill the knowledge gap in this area. Using qualitative research methods, we investigate the relationships between UGS and healthy ageing in two megacities in India.

## 2. Methods

### 2.1. Study Context and Methods

We focused on two, million-plus (population of 1 million or more), urban agglomerations in India. Between May 2019-January 2020, participants (*N* = 60, female = 51%) were recruited from the cities of New Delhi (population = 18.6 million) and Chennai (population = 10.9 million). A snowball sampling technique with multiple recruitment approaches was adopted to reach out to participants. A gender-and age-sensitive approach was employed to ensure equal representation of men and women above 60 years of age in this study [22]. In each city, the lead researcher (DA) commenced with a small population of known individuals and expanded the sample by asking the initial participants to identify others that were interested in participating in the study. The lead researcher was supported by one male and one female bilingual research assistant in each city. Research assistants had prior experience in qualitative research methods and were required to participate in a training session on roles and responsibilities, interview techniques, dealing with sensitive questions, data protection codes of conduct and practicalities of field work. The lead researcher (DA) supervised all research assistants and served as the primary source of contact for all queries related to this study.

Recommendations from community gatekeepers such as resident welfare associations, neighbourhood watch groups, and resident associations were used to identify an initial set of eligible participants. Following this, snowball sampling was used to recruit a larger sample. From their social networks, participants recommended other older adults who were willing to participate. Recruitment activities also included canvassing in study neighbourhoods and during social activities hosted by apartment housing blocks and neighbourhood associations. Snowball sampling has been recommended by researchers who have conducted similar studies in LMIC contexts to enable the selection of individuals that are especially knowledgeable about the variables of interest [23]. This approach reinforced the depth of understanding of the lived experiences of older adults in their immediate surroundings [24].

Eligible participants were contacted either in-person or via telephone and invited to participate in the study. Eligibility criteria for this study included: (i) currently residing in the city of New Delhi or Chennai; (ii) residents of the city for at least 6 months; (iii) ≥60 years of age; (iv) able and willing to communicate in the official languages in the study regions—English, Hindi or Tamil; (v) no visible signs of inappropriate or disruptive behaviour (e.g., insobriety, substance abuse). An international advisory group from the International Physical Activity and Environment Network (IPEN; www.ipenproject.org) was consulted during the study stages to ensure scientific validity and integrity of study protocols and offer triangulation of the results [25]. This process was used to resolve any disagreements and reach a consensus for coding and data analysis. We use the consolidated criteria for reporting qualitative research (COREQ) checklist for reporting our qualitative research methods and findings (Supplementary Materials) [26].

### 2.2. Data Collection

Between August 2019 and February 2020, semi-structured interviews were conducted with participants (Chennai n = 32, New Delhi n = 28) using an interview guide that was developed for this study (Supplementary Materials). The interview guide included open-ended questions that encouraged participants to speak freely about what they felt were relevant and important in terms of their home and family setting, features in the built environment, quality of neighbourhood parks and UGS, work, leisure and travel behaviours, modes of transportation, and social interactions in the community. Among eligible participants that were approached, six participants refused to participate in the study citing illness.

Information about the study, including interview questions and consent forms, were provided to all participants before the scheduled interview. Participants were able to select their interview location and language preference to ensure their comfort and convenience. Most interviews were conducted in a private room in participants’ homes and lasted between 45–60 min (mean = 52 min). A conversational style was adopted by the interviewee and the interview guide was used to navigate the conversation. Researchers made comprehensive field notes during and after the interview detailing the overall setting to provide rich context of the study. Field notes included basic information on the study title, name of the researcher, date of interview, season of data collection and pertinent information about the weather. Field notes also included information on cultural norms, family values, customs, and aspects related to the geographic setting or locations of features relevant to the study topic of interest.

All interviews were digitally recorded and transcribed verbatim. The transcribed text was anonymized to maintain the privacy and confidentiality of participants. Theoretical saturation of data—a situation where no new information, concepts or themes were emerging from the data—was used to determine the number of participants required for a detailed analysis. There are no fixed sample sizes or standardized tests to estimate the amount of data needed for achieving saturation [27]. All study procedures were approved by the Research Ethics Committee at Queen’s University Belfast, UK (approval file number EPS 19_224).

### 2.3. Data Analysis

Interviews conducted in Hindi and Tamil were translated to English by a bilingual person knowledgeable of the common cultural terms and linguistic expressions used in New Delhi and Chennai respectively. To ensure data reliability and rigour, translations were evaluated by two bilingual linguistic experts who were not familiar with the project. Interviews were analysed by a process of inductive and thematic analysis using NVivo 12 (QSR International Pty Ltd., 2018) [28,29].

A nested coding structure was developed by the research team and data was categorised into key themes by identifying and interpreting emerging patterns of meaning or common constructs within the qualitative data. Inductive analysis was conducted in the following phases: (a) data familiarisation, (b) division of interview text into meaning units, (c) grouping of meaning units, (d) formulation of descriptive labels and codes, and (e) development of categories and themes. Codes and labels were compared and appraised to determine which codes seem to belong together, thereby forming a category or theme [30,31]. All interview transcripts were analysed and independently coded by two members of the research team for consistency. The coding structure was validated with the wider research team to refine and agree on codes. Overlapping aspects of coding and categorisation of hard to place data was discussed with the advisory team. A summary report of themes and findings was shared with each participant and they were provided with an opportunity to provide feedback.

## 3. Results

### 3.1. Sample Characteristics

Study participants ranged in age between 60 and 78 years (median age = 67 years), of which half (*n* = 31, 51.7%) were female. The largest proportion (*n* = 48, 80%) were living with someone, and a fifth of participants (*n* = 12, 20%) were living alone, with 66.7% (*n* = 40) retired, and a third (*n* = 20, 33.3%) employed. Sample characteristics are shown in Table 1.

Key themes on the role of UGS in healthy ageing emerged through the process of inductive analysis. Participants discussed the benefits of UGS, park quality, and constraints to active living. Some constraints were unique to the individual and their immediate neighbourhoods, but a majority were shared by participants in both cities. Four key themes emerged from the interviews (Table 2): (1) health benefits of UGS; (2) quality of UGS; (3) community building and social capital; and (4) benefits for disadvantaged groups.

### 3.2. Health Benefits of Urban Green Space

Findings highlighted the role of UGSs for physical activity promotion. Walking was reported as the most common behavior and park walkways were the most commonly used physical activity areas by older adults in both cities. Older adults with reduced mobility preferred UGSs that were easily accessible and near their homes. Due to intense urbanisation and rapid growth in motorised traffic, a lack of safe walking infrastructure emerged as a common barrier in both cities.

*“I have diabetes and the doctor has asked me to go for a brisk walk for 30 minutes every day. I live in a very congested neighbourhood and I do not drive, so this park is my only option as it is a five-minute walk from my home.”* (Male, Age 62, Chennai)

*“It is impossible to walk on the footpaths. They are poorly designed. Many uneven surfaces and also a high step from the road. The park is nicer and safer. I can walk here without worrying about being hit by a car”* (Female, Age 65, New Delhi)

*“This park has something for everyone. There is a play area and sandpit for children, a yoga pavilion, a circular walking pathway, outdoor gym equipment, and some play courts. We are lucky to live here; very few neighbourhoods have the luxury of any green space anymore.”* (Female, Age 65, Chennai)

Participants reported lower levels of exposure to noise, air pollution, and heat in UGSs. For many older adults, UGSs offered respite and escape from toxic exhaust fumes from motorized vehicles and polluted air in the city.

*“This park gives me the only chance to breathe clean air. Being here is freedom from the dust and pollution in Delhi.”* (Male, Age 66, New Delhi)

*“I am at peace when I am here. It is a break from the exhaust fumes of traffic and constant noise of honking vehicles and construction happening near my house.”* (Female, Age 63, New Delhi)

### 3.3. Quality of Urban Green Spaces

Participants reported that access routes between home and UGSs were not designed and maintained effectively. In some neighbourhoods, the design of park features, pedestrian infrastructure, and the streetscape did not meet the needs of older adults with mobility difficulties. Lack of safe walking infrastructure (e.g., unobstructed footpaths, non-slip surfaces, street lighting) and age-friendly facilities (e.g., benches, toilets) in parks were barriers to the use of UGSs.

*“I like to go to the park at the end of my street, but it is difficult because it does not serve my needs. I suffer from joint pain and need to sit down frequently. There are no resting spots, benches, or toilets, so I find it difficult when I am there.”* (Male, Age 72, New Delhi)

*“The park in my neighbouhood is very popular with children and younger people because it has many facilities for them. There is a badminton court and volleyball net. But, for older people like me, there is nothing. Not even proper benches with a backrest and arms or non-slip surfaces to walk. When it rains, it is so risky. One of my friends slipped and hurt herself.”* (Female, Age 65, Chennai)

*“The footpaths are no longer places where people can walk. They have become parking bays for cars. Without the park, I don’t know where I would walk safely and go for exercise. It is impossible to walk on the streets these days. They are filled with motorcycles, cars, autorickshaws leaving no space for us.”* (Female, Age 66, Chennai)

Participants attributed the lack of parks and diminishing street trees to the rapid rates of urbanisation, increased traffic, and congestion on roads. Green space was more important to older adults living in high-rise apartments and multi-occupancy residential dwellings.

*“The streets in my area used to have these old banyan trees which used to give shade. It was a pleasure to walk here. Over the past 50 years, construction and development have destroyed them. It is impossible to walk here in the scorching heat. There is no sign of nature here now, so I don’t go out for walks like I used to.”* (Male, Age 66, Chennai)

*“In the hot summer months, it is impossible to go anywhere. The park is the only green space in a jungle of concrete. It is like a place of refuge for many in my building.”* (Male, Age 62, New Delhi)

### 3.4. Community Building and Social Capital

UGSs provided a neutral space for social interactions to occur and where people from different cultures and backgrounds could come together. Participants described new friendly relationships or partnerships developing as a result of impromptu and unplanned interactions in parks. While personal goals or desires were achieved, community building and increased social capital also emerged. Individual and community benefits, improved health, and social resilience were reported as key outcomes.

*“I have been living alone for the past few years. This park has given me an outlet and a chance to meet neighbours during my evening walks. I have made friends here and they check on me if they don’t see me outside for my walk. It gives me comfort knowing that I have a community nearby.”* (Male, Age 70, New Delhi)

*“I have a big extended family and bring my grandchildren to this park. The big advantage is that there is a play area for kids and a few benches for older people like me to sit. I have managed to meet other grandparents and we have a nice community now. All our grandchildren play with each other.”* (Female, Age 67, Chennai)

Community gardens created avenues for informal gathering older adults took ownership of the space, collectively grew plants, and developed friendships. Some participants reported working on a community project or common goal with others in the neighbourhood.

*“There was a neglected corner of the park that was becoming a garbage dump. One of my friends decided to clean it up and plant shrubs and flowers. I also joined her, and then many others joined us. We now take turns in watering the plants. It has become a collective effort.”* (Female, Age 66, New Delhi)

*“My neighbour and I started a community garden near the park. We grow vegetables, herbs, and flowers. We also started a small gardening group where we share tips and exchange plants with others. This has now grown to over 75 participants. It has given me something to look forward to and keeps me busy.”* (Female, Age 60, Chennai)

Social and cultural events were often held in parks and gardens, providing opportunities for older adults to interact and develop social bonds.

*“There is a shrine in the centre of the park managed by the neighbourhood association. Every morning, there is a puja (an act of worship) attended by many park visitors. Many people bring flowers and garlands they have made themselves and also make rangoli (decorative floor patterns). On auspicious days and festivals like the New Year or Diwali, we have a big gathering here and everyone celebrates together.”* (Male, Age 62, Chennai)

*“On Children’s Day, a group of us… retired grandparents organise outdoor cultural events for children in the park. Last year, we had face painting and many outdoor games like hopscotch and tug of war.”* (Female, Age 65, New Delhi)

### 3.5. Benefits for Socio-Economically Disadvantaged Groups

Low-income populations in both cities reported lower access to UGS and parks with lower quality, maintenance, and safety than high-income residents. People living in socio-economically disadvantaged neighbourhoods recognised and appreciated the value of UGSs, but the most convenient spaces were often of poor quality.

*“I live in public housing in very cramped conditions. There are small streets in a bad condition, open drains, poor lighting. The streets are too small to even have trees around them. We do not have space inside our homes, so the playground is the only space to get a breath of fresh air. I wish the municipality would improve its quality. Everything is broken and damaged. Even a few benches for older people to sit will help.”* (Male, Age 64, New Delhi)

*“I used to work as a housekeeper and was very active until I was 60, but then I had a stroke and one side of my body was affected. The doctor recommended physiotherapy, but I could not afford it long-term. The gym equipment in the nearby park is the only free option I have to exercise, but some of it is broken and unusable.”* (Female, Age 62, Chennai)

Neighbourhood disorder (e.g., vandalism, graffiti, litter) constrained older adults’ use of UGS. Older women, in particular, reported feeling unsafe while walking through UGSs and vulnerable to anti-social behaviour. Some older adults avoided UGSs because of crime, signs of physical disorder, and threats to personal safety.

*“The park is only used by groups of men to drink alcohol. It is not safe for women so I avoid it. They are always loitering around, hanging around at the entrance to the park. I don’t even feel like going there. It is so unpleasant.”* (Female, Age 62, New Delhi)

*“I work as a cleaner all seven days of the week. Sometimes, I walk through the park which is on the way, but there is no proper lighting on the paths. When I leave for work in the mornings, it is still dark and most of the time, the street lights are not working, or someone has broken them. This is the only chance I have to walk in a green space and escape from the traffic and noise, but I don’t feel safe in the park.”* (Female, Age 61, Chennai)

## 4. Discussion

Our study highlights the relevance of UGSs for healthy ageing in rapidly urbanising megacities in India. Predominantly, research investigating the links between UGS and health have been conducted in HICs. Our research draws from the lived experiences of older adults in two high-density cities in India and examines the influence of UGS on behaviours that promote healthy ageing, which is fundamental for the design of age-friendly cities. We explored several pathways through which UGS may promote positive health, social and environmental outcomes, which are consistent with previous research in HICs [32,33]. UGS may promote mental and physical health in older adults by providing opportunities for psychological relaxation and stress alleviation, supporting physical activity, stimulating social cohesion [5,33]. UGSs may also reduce exposure to harmful factors which older adults are especially vulnerable to such as toxic air pollutants, noise, and excessive heat.

Access to UGS provided an incentive for everyday physical activity and improved social interaction, which is especially important for older women. In our study, women reported participating in group-based physical activity programmes and community projects with new friends as a result of increased social interactions in their local UGS. Studies have found that women who frequently exercised in a group developed a sense of familiarity with each other and therefore felt safer [34]. This is especially important in India, as women over the age of 60 are the fastest-growing age group with insufficient physical activity levels and higher levels of disability compared to men [35].

Neighbourhoods with higher levels of UGS foster social cohesion and reduce feelings of loneliness, which are key predictors of health in the older adults [36,37]. In our study, participants reported increased communication and non-familial intergenerational interaction between younger and older adults in UGSs. Successful non-familial intergenerational interactions were achieved through shared experiences and meaningful outdoor activities in UGSs. Strong social connections are linked to lower rates of early mortality, less fear of crime, reduced loneliness, and better physical health in older adults [38].

Our study demonstrated the transformation of community liabilities into assets. In both cities, community gardens were established through informal interaction with neighbours and social networks. Studies of the social dynamics of community gardens have illustrated the relationships between urban greening and community building [39,40]. Grassroots initiatives such as community gardens can transform dilapidated vacant lots into usable UGSs. Once created, these UGGs can continue to strengthen social ties and build a sense of community.

For UGSs to benefit the health of older adults, the quality and design of the features may need to be adapted. In our study, features such as benches, shaded walking paths with non-slip surfaces and outdoor lighting were especially important. For older adults to move around, walkability, connectivity, and safety of streets around the UGS, and access to public transport are key factors. The quality of UGS is dependent on its integration with neighbourhood planning, design of streetscapes, and pedestrian infrastructure [5,6].

Results from this study underscore the fact that everyone can benefit from UGS, but they can be of particular relevance for older adults living in socially disadvantaged or underserved communities, which often have the least access to high-quality parks and green spaces. Neighbourhoods with high deprivation and crime rates, deficient quality of shelters and housing stock, overcrowding, poor sanitation, and lack of access to healthcare services can exacerbate health and well-being in later life [41]. In such areas, outdoor environments like parks and UGS can play a key role in enhancing physical and mental health, maintaining social networks, and fostering a sense of belonging among older adults.

### 4.1. Strengths and Limitations

The strength of this study was the use of qualitative research methods to investigate the topic of interest and the use of inductive and thematic analysis. Using exploratory techniques with participants sharing narratives of lived experiences and in-built mechanisms to ensure the reliability of data and validity of thematic analysis enabled a richer understanding of the topic. Independent researchers analysed the data and the expertise of the advisory group was used to harmonize discordance and create consensus, ensuring rigor and consistency. This process highlighted the importance and relevance of including qualitative data to understand the mechanisms for a specific population of interest. The sampling and site selection in two megacities cities located in the north and south India captured cultural diversity. To our knowledge, this is the first study on the role of UGS for healthy ageing in India.

Several limitations of this study must be considered. The modest sample size and the non-probability nature of the sampling strategy may reduce the generalisability of findings. Drawbacks of qualitative interviews include reliance on respondents’ accuracy and their intensity in terms of time, expense, and possible emotional strain. The analysis did not account for socio-demographic characteristics of participants and is therefore insufficient to make population-level summaries. This study was conducted in two megacities in India which may restrict environmental variability. Caution should be exercised before generalising findings to populations that are different from the study settings, for example much smaller urban and rural areas in India. Findings are not representative and cannot be extended to wider populations. Delhi and Chennai have dissimilar UGSs, which have diminished over time. The study did not capture information on the availability of UGS per site which could differentially impact the experience of UGS in study participants. This points to the need for care in extrapolating the results beyond the study areas.

### 4.2. Implications for Research, Practice, and Policy

The introduction of more UGS in urban settings has been widely endorsed as a method to improve both physical and mental health in older adults. Recent research efforts in healthy ageing in LMICs have begun to fill important research gaps. However, the knowledge provided by these studies is limited by study design and data quality. A consideration of contextual, cultural and environmental factors mediating the relationship between UGS and health outcomes is necessary. For example, future research in LMICs should assess cultural norms, traditions, and acceptability of physical activity and recreation in UGS among certain populations, especially women. An understanding of factors such as local climate, heat exposure, environmental pollutants, crime and safety that may be compromising the quality and access to UGS is necessary in LMICs [42]. Longitudinal studies that use objective indicators of UGS, built environment audits, and health indicators are also warranted.

Researchers should be included as policy experts in panels of state and central government urban development ministries and urban planning agencies. Creating age-friendly communities will require planners to collaborate with practitioners and researchers from other disciplines and integrate public health theories and practices into planning pedagogy. There is a need for better inclusion of health and equity outcomes in planning UGSs, improved monitoring and management of local UGS in India.

Understanding how to design, deliver, and manage UGSs effectively is essential for creating age-friendly communities. UGSs seem to be most effective when the physical design of the green space is coupled with community engagement and participation elements. The provision and administration of high-quality UGS intertwine issues of planning, design, management, and governance [43,44]. Local decision-makers and planners will need to collaborate to address inequities in UGS provision and implement mitigation measures. Examples include the protection of environmentally sensitive areas, promotion of urban agriculture, biodiversity enhancement through tree planting and native landscaping, and prioritization of UGS in socio-economically disadvantaged communities [45].

## 5. Conclusions

Indian cities are currently facing serious threats due to environmental degradation, increasing carbon emissions, air pollution, and temperature (urban heat island effect) [45,46]. In dense megacities in India, uncontrolled expansion, poor accessibility and connectivity to neighbourhood parks, diminishing UGS, and biodiversity are pressing challenges to environmental sustainability and health of ageing populations. As health disparities are increasingly understood as a product of social, economic, political, and physical inequalities in places, urban planners will need to play a more active role in understanding and addressing these inequities. Multidisciplinary, cross-sectoral collaborations and community engagement between the fields of urban planning and public health are essential to ensure that UGSs provide functional opportunities for older adults in LMICs.

## Figures and Tables

**Table 1:** Descriptive characteristics of study participants (*N* = 60).

Age (in years)	Median (SD)
	67 (4.47)
Location	n, %
New Delhi	28 (46.7%)
Chennai	32 (53.3%)
Gender	n, %
Male	29 (48.3%)
Female	31 (51.7%)
Marital status	n, %
Married	38 (63.3%)
Not married/widowed	22 (36.7%)
Living situation	n, %
Living with someone	48 (80.0%)
Living alone	12 (20.0%)
Employment status	n, %
Retired	40 (66.7%)
Employed	20 (33.3%)

**Table 2:** Summary table of themes and sample quotes (*N* = 60) highlighting perspectives on urban green spaces and ageing in New Delhi and Chennai, India.

Themes	Quotes
**Health benefits of urban green space**	*“The roads are busy and pavements are always crowded and chaotic. They are too many hurdles and no continuous flat surface. I have to constantly climb up and down from the footpaths as they end abruptly. The park is the only safe space since it allows me to walk without worrying about falling or motorbikes and traffic hitting me.”*—Male, Age 66, Chennai
	*“The pollution in Delhi is unbearable. At home, we have air filters, but when I step outside, I usually walk in the neighbouhood park. At least there are some trees and plants there which provide fresh air and shade, otherwise on the streets, I am only breathing the fumes from vehicles and suffering under the hot sun” *—Female, Age 69, New Delhi
**Quality of urban green space**	*“The park near the signal is the only open space for everyone in this crowded area. It gets very busy in the morning and evening, so I come early to avoid the crowds.”*—Male, Age 62, Chennai
	*“The park in my neighbouhood and footpaths leading to it are full of garbage and litter. It is unpleasant to walk there and puts me off, so I don’t go there.”*—Female, Age 71, New Delhi
	*“I have arthritis and the doctor has told me to exercise my knees and ankles. But I live in this busy and crowded area where there are many shops. There is no space to walk on the footpaths because they are crowded. Sometimes my son will drive me to the park, but otherwise I stay inside the flat”*—Female, Age 74, New Delhi
	*“I cannot walk without support, so I do not like to go outside the house. If someone takes me to the park, them I go, but otherwise, I am worried about falling on the uneven footpaths.”*—Female, Age 76, Chennai
**Community building and social capital**	*“I have made new friends here and we meet in the park for yoga every morning. I like doing that because other people are around. Some of us also come here for evening walks. Because I am alone at home all day, I find it is nice to do these in a group.”*—Female, Age 64, New Delhi
	*“I have knee pain and have been advised to walk everyday. I started coming to this park every evening and met others who also have health issues. We have formed a walking group and it keeps us motivated.”*—Male, Age 78, Chennai
	*“The park provides something for everyone. We have a temple in the park and the regular park visitors have formed a management committee. They take turns in supervising the cleaning, decoration and morning prayer rituals at the temple. There is another committee that oversees the yoga and exercise classes. It has brought the community together.”*—Male, Age 75, Chennai
**Benefits for socio-economically disadvantaged groups**	*“I bring my grandchildren here to play. This park is the only free and safe outdoor space near our home where they can run around and play.”*—Female, Age 62, Chennai
	*“I drop my grandson to school everyday and we walk through the park to avoid the traffic and pollution. This section of the walk is the only highlight, otherwise the constant noise and fumes from traffic are horrible.”*—Male, Age 66, New Delhi

## Data Availability

The data analyzed in this study are available from the corresponding author upon request.

## Supplementary Materials

The following are available online at https://www.mdpi.com/1660-4601/18/4/1491/s1.
