# Supplementary material for: Designing Age-Friendly Communities: Exploring Qualitative Perspectives on Urban Green Spaces and Ageing in Two Indian Megacities
Source: Int J Environ Res Public Health. 2021 Feb 4;18(4):1491. doi: 10.3390/ijerph18041491 (PMC7914589; doi:10.3390/ijerph18041491)
Supplement: Supplementary file 1 [file ijerph-18-01491-s001.zip › Appendix1.docx]

**Appendix 1. Interview transcript for study on urban green spaces and ageing in New Delhi and Chennai, India.**

Introduction: Thank you for agreeing to participate in this interview. We will be talking with older adults across the city and requesting your views on parks and green spaces. The interview will take about 45-60 minutes.

When we talk about home neighborhood, we mean the area around where you live.

1. Think about where you live. How often do you walk/ bicycle in your neighbourhood? (Prompts: everyday, few times every day, few times during a week)
2. Can you describe the street and footpath conditions in your neighbourhood? (Prompts: crosswalks, obstructions, trees, shade, safety, traffic)
3. Do you see your neighbors/ other people walking in your neighbourhood? If yes, at what times of the day?
4. How easy or difficult is it to get to neighbourhood open spaces such as parks, playgrounds or other green spaces?
5. What is the quality of the open space(s)? Are there any safety or appearance concerns in the neighbourhood surrounding the open space? (Prompts: poor lighting, graffiti, vandalism)
6. How many points of entry does the neighbourhood open space(s) have? Is there a public transit stop within sight of the neighbourhood open space(s)?
7. Can you describe the main land use(s) around the neighbourhood open space(s)? (Prompts: Residential, Commercial, Institutional (e.g., school), Industrial (e.g., warehouse))
8. What is the quality of pedestrian or cycling infrastructure around the neighbourhood open space(s)? Are there sidewalks or bicycle paths on any roads bordering the neighbourhood open space(s)? Is there an external path or trail connected to the open space(s)?
9. Can you describe the amenities or facilities inside the neighbourhood open space(s)? (Prompts: public toilets, drinking water, benches, picnic tables, dustbins)
10. How is the aesthetic quality of the neighbourhood open space(s)? (Prompts: landscaping, trees, artistic features such as statues, sculptures water features)?
11. Can you describe what you do when you visit the neighbourhood open space(s)? What types of activities do you participate in? (Prompts: walking, bicycling, playing sports, yoga, interacting with others)
12. Think about any other local places you regularly travel to. Can you describe your commute to these places; for example, commute to work, to shop, to a nearby park, for errands? (Prompts: workplace, school, errands, shopping, parks, place of worship, walk, bike, public transport) How long do you spend commuting to each of these places?
13. What modes of transport do you use while commuting? Does any portion of your commute include walking, for example, walking to a bus stop, train station? (Prompts: walk, bicycle, car, bus, suburban train, a combination of these modes)
14. Do you have good and bad commute days? What makes a good commute? What makes a bad commute? (Prompts: good—little traffic, fair weather, bad—traffic accidents, rain)
15. What are some of the challenges/ obstacles you encounter when you walk/ bicycle/ commute?

(Prompts: congestion, damaged sidewalks, trash, illegally parked cars and motorbikes, vendors, road signs, construction debris) How do you address these challenges?
