# Supplementary material for: Designing Age-Friendly Communities: Exploring Qualitative Perspectives on Urban Green Spaces and Ageing in Two Indian Megacities
Source: Int J Environ Res Public Health. 2021 Feb 4;18(4):1491. doi: 10.3390/ijerph18041491 (PMC7914589; doi:10.3390/ijerph18041491)
Supplement: Supplementary file 1 [file ijerph-18-01491-s001.zip › Appendix2.docx]

| Appendix 2. A checklist for comprehensive reporting of qualitative studies adapted from Tong A, Sainsbury P, Craig J. (2007) | | |
| --- | --- | --- |
| **Item number** | **Guide questions/description** | **Reported on page #** |
| **Domain 1: Research team and reflexivity** |  |  |
| 1. Interviewer/facilitator | Which author/s conducted the interview? | 3 |
| 2. Credentials | What were the researcher’s credentials? | 1 |
| 3. Occupation | What was their occupation at the time of the study? | 1 |
| 4. Gender | Was the researcher male or female? | 3 |
| 5. Experience and training | What experience or training did the researcher have? | 3 |
| 6. Relationship with participants established | Was a relationship established prior to study commencement? | 4 |
| 7. Participant knowledge of the interviewer | What did the participants know about the researcher? | 4 |
| 8. Interviewer  characteristics | What characteristics were reported about the interviewer/facilitator? | 4 |
| **Domain 2: study design** |  |  |
| 9. Methodological  orientation and Theory | What methodological orientation was stated to underpin the study? | 5 |
| 10. Sampling | How were participants selected? | 4 |
| 11. Method of approach | How were participants approached? | 4 |
| 12. Sample size | How many participants were in the study? | 4 |
| 13. Non-participation | How many people refused to participate or dropped out? Reasons? | 4 |
| 14. Setting of data  collection | Where was the data collected? | 4 |
| 15. Presence of non-  participants | Was anyone else present besides the participants and researchers? | 4 |
| 16. Description of sample | What are the important characteristics of the sample? | 6 |
| 17. Interview guide | Were questions, prompts, guides provided by the authors? | Appendix 1 |
| 18. Repeat interviews | Were repeat interviews carried out? | 4 |
| 19. Audio/visual recording | Did the research use audio or visual recording to collect the data? | 4 |
| 20. Field notes | Were field notes made during and/or after the interview? | 4-5 |
| 21. Duration | What was the duration of the interviews | 4 |
| 22. Data saturation | Was data saturation discussed? | 5 |
| 23. Transcripts returned | Were transcripts returned to  participants for comment and/or correction? | 5 |
| **Domain 3: analysis and**  **findings** |  |  |
| 24. Number of data coders | How many data coders coded the data? | 5 |
| 25. Description of the  coding tree | Did authors provide a description of the coding tree? | 5 |
| 26. Derivation of themes | Were themes identified in advance or derived from the data? | 5 |
| 27. Software | What software, if applicable, was used to manage the data? | 5 |
| 28. Participant checking | Did participants provide feedback on the findings? | 5 |
| 29. Quotations presented | Were participant quotations presented to illustrate the themes/findings? Was each quotation identified? | 5 |
| 30. Data and findings  consistent | Was there consistency between the data presented and the findings? | 6-8 |
| 31. Clarity of major  themes | Were major themes clearly presented in the findings? | 10, Table 2 |
| 32. Clarity of minor themes | Is there a description of diverse cases or discussion of minor themes? | 5 |

Developed from: Tong A, Sainsbury P, Craig J. Consolidated criteria for reporting qualitative research (COREQ): a 32-item checklist

for interviews and focus groups. International Journal for Quality in Health Care. 2007. Volume 19, Number 6: pp. 349 – 357
